# A review of factors affecting the success of geminivirus infectious clones

**DOI:** 10.1007/s00299-025-03560-4

**Published:** 2025-08-04

**Authors:** S. R. Möller, M. N. Maruthi

**Affiliations:** University of Greenwich, Natural Resources Institute, Medway, Kent UK

**Keywords:** Agrobacterium infiltration, Begomovirus, Plant immunity, Biolistic, Virus transmission

## Abstract

**Key message:**

**In this review, we provide a summary of factors that affect the successful infection of geminivirus clones in plants to enable the greater understanding of plant–virus interactions.**

**Abstract:**

Geminiviruses are single-stranded DNA viruses that can cause significant losses in economically important crops worldwide. Considerable efforts have been made to study the geminiviruses in detail, which has resulted in the construction of many infectious clones for the vast diversity of geminiviruses. In laboratory conditions, agrobacterium or occasionally biolistic methods are used to deliver viral DNA to the plant cell. However, not every delivered viral DNA will develop into an infection due to several reasons. In this manuscript, we review the factors that affect the success of geminivirus infectious clones. Factors affecting virus infection including the methods of inoculating in vitro-generated viral DNA constructs are often neglected, leading to failed virus infections and drawing wrong conclusions. Deciding exactly where on the plant to inoculate, what age of plant, and what agrobacterium strain are all examples of variables which may influence an infection. We find that stem injections of agrobacterium into young seedlings with an optical density at 600 nm (OD_600_) in the 0.1–0.3 range are an optimal starting point for studies. This review will provide a thorough compilation of inoculation methods and use this to discuss the deeper mechanisms at play during the initial infection of plants with geminivirus infectious clones.

**Graphical abstract:**

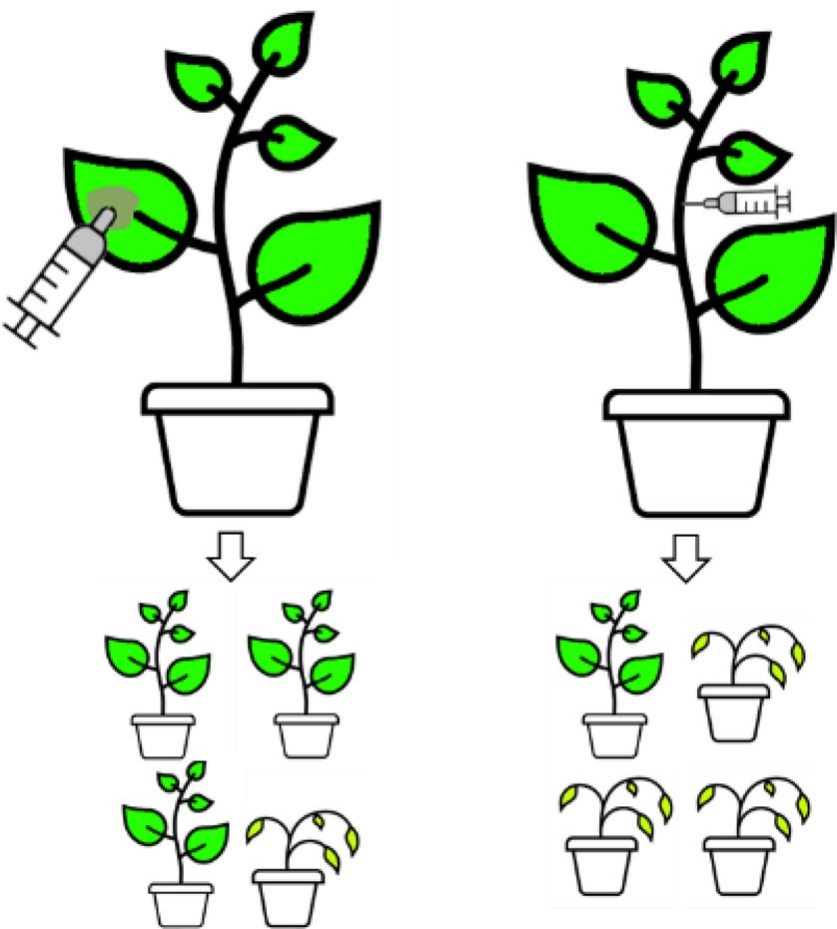

## Introduction

**Fig. 1:**
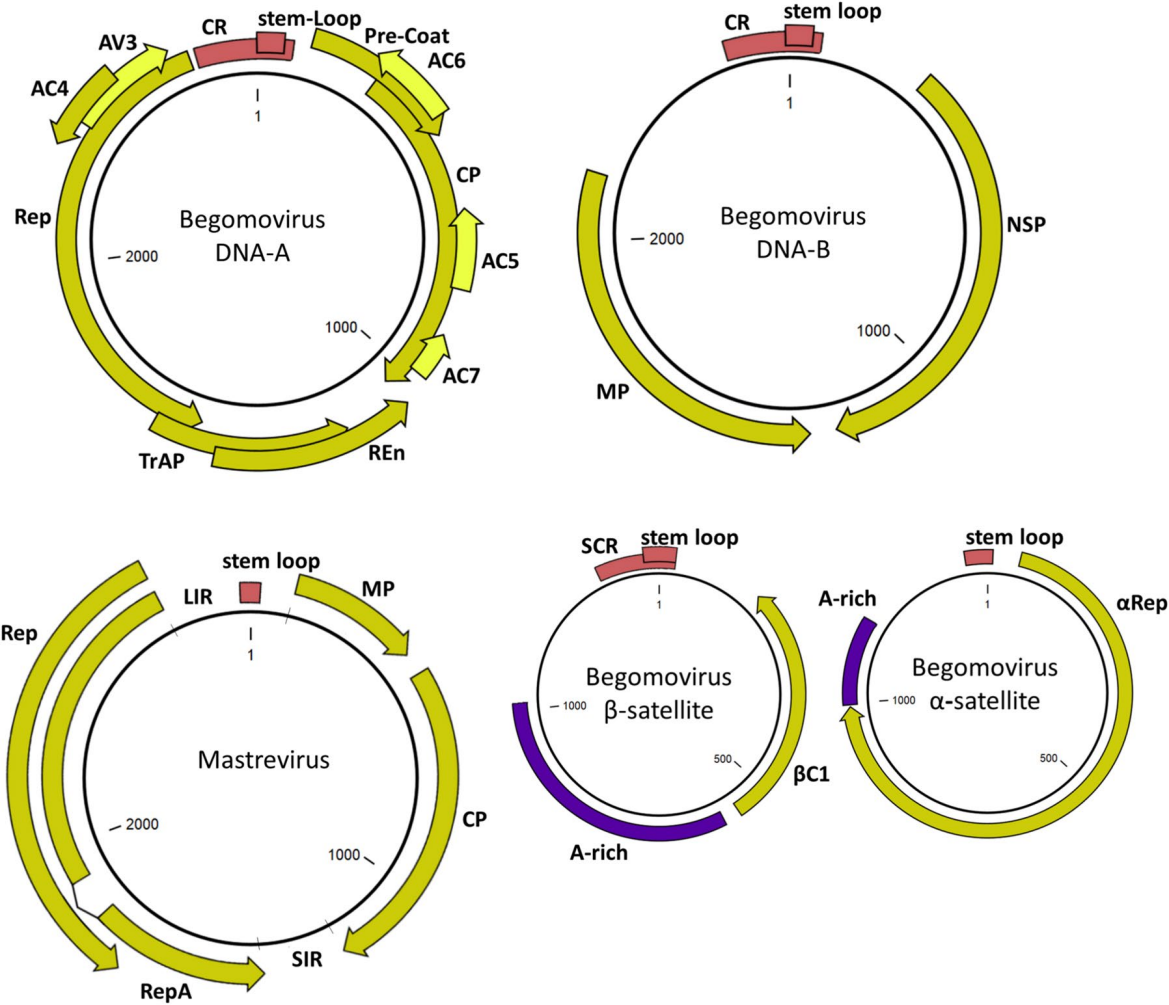
Typical structure of geminiviral genomes (only begomovirus and mastrevirus genomes shown as examples). The common region (CR) contains replication origin. Rep replication protein—in mastrevirus, the splice variant RepA is present which takes on additional functions similar to TrAP and REn (Diamos and Mason 2019; Watanabe and Ugaki 2021); TrAP Transcription activator protein; REn replication enhancer; SIR Small intergenic region which acts as terminator; CP coat protein; Pre-CP pre-coat protein which also aids in movement; V1 coat protein; AC4 is a virulence factor; MP movement protein; NSP nuclear shuttle protein; SCR satellite conserved region; βC1 satellite pathogenicity factor; A-rich adenine rich region. Reading frames AC5, AC6 and AV3 are additional small reading frames potentially found in begomoviruses with additional putative functions *e.g.* in silencing suppression(Gong et al. 2021; Wang et al. 2022b)

Geminiviruses are a group of plant viruses that can infect and cause diseases on a wide range of both dicot and monocot species in the tropics, causing high economic losses, sometimes up to 100%, at least since the 1970s, in several food, fibre, fruit and industrial crops such as beans, cassava, cotton, maize, tobacco and many vegetables (Rojas et al. 2018). Family *Geminiviridae* is the largest group of single-stranded DNA viruses infecting plants, currently containing about 520 distinct species. The main genus *Begomovirus* in the family *Geminiviridae* contains at least 445 distinct species, whilst the second largest genus *Mastrevirus* contains about 45 species. The remaining 30 species are spread out amongst the 15 other genera with only a few viruses in each (Fiallo-Olivé et al. 2021; Roumagnac et al. 2022). Geminiviruses derive their name from their coat protein (CP) structure which takes the form of twinned icosahedral particles. Each particle contains a single copy of a single-stranded circular DNA molecule of around 2600 to 3200 base pairs in length (Hesketh et al. 2018). Most geminiviruses are monopartite with a single-genome DNA-A containing 4 to 8 overlapping open reading frames (ORFs) or genes. However, some begomoviruses are bipartite and have an additional DNA-B component, which codes for two ORFs. In addition, smaller satellite DNAs are found in association with some geminiviruses (Zhou 2013; Briddon et al. 2018). Geminiviruses confine to the phloem and are often found in fully differentiated cells; hence, the virus must carry out some reprogramming of the cell cycle to acquire the factors needed for replication (Hanley-Bowdoin et al. 2013). The genome structure of geminiviruses (Fig. 1) differs slightly between the various genera. Common to them is the presence of an intergenic region containing the iteron sequences, bi-directional promoters and a stem-loop structure required for DNA replication. The principle of making infectious clones remains the same for all geminiviruses; the virus must contain slightly more than one unit length which encompasses two stem loops, which allows it to reform into circular DNAs (Saad et al. 2021). This also extends to beta-, alpha- and delta-satellites as well as to other circular DNA viruses such as nanoviruses. Among the ORFs, geminiviruses encode a replication protein (Rep), which does not have its own DNA polymerase activity but instead recruits host polymerases to replicate the virus. The Rep cleaves the, mostly, conserved nonanucleotide motif TAATATTAC. The Rep cleaves a single strand of DNA, favouring the viral sense strand, and facilitates the rejoining to initiate virus replication (Laufs et al. 1995). Replication was initially considered to be rolling circle replication where the Rep binds to iteron sequences in the origin of replication (*ori*) and recruits host polymerases to continuously copy the genome (Saunders et al. 1991). Later, electron microscopy and further analysis of the replication intermediates suggested a recombination-dependent virus replication method, whereby heterologous double-stranded linear fragments are produced which then recombine to form circular DNA (Jeske et al. 2001). This discussion is reviewed in depth in Bonnamy et al. (2023). For geminiviruses, the Rep proteins and virus factors for cell cycle reprogramming are normally transcribed from the complementary sense strand, whilst the CP and proteins involved in cell-to-cell movement are normally transcribed from the viral sense strand. Most geminiviral proteins carry out multiple functions by interacting with several distinct host factors to promote a cellular environment supporting viral infection, suppress silencing mechanisms and facilitate movement. This is reviewed in Fondong (2013) and Fiallo-Olivé et al. (2021).

As new techniques have made cloning viral vectors easy (Ferro et al. 2019; Yıldırım et al. 2023), achieving efficient initial infections in relevant crop plants has often remained a possibility (Liu et al. 2021; Zhang et al. 2024; Kavalappara et al. 2024; Kumari et al. 2024).

Geminivirus particles are naturally introduced to plants by insects, which have acquired the virus from feeding on infected plants. The difficulties, however, is the maintenance and handling of insects in laboratory conditions, which give rise to issues with insect containment and contamination. Alternative methods of virus inoculations, therefore, using *A. tumefaciens* (henceforth referred to as agrobacterium) wounding, and/ or particle bombardment have been sought. Collectively, geminiviruses infect a vast range of plant hosts, but individual viruses can have a narrower host range. Even minor variations within the virus can make it ineffective in a host plant (Vo et al. 2023), and variants of plants form the basis of resistant cultivars. One might anticipate a geminivirus to produce infections in all the inoculated plants of a susceptible species. Yet in many cases, only some of them develop an infection following the inoculation and thus creating uncertainty on the use of infectious clones in experiments. Having a high percentage of inoculated plants become symptomatic is of interest to, for example, screen for resistant crop varieties and understand the host range of viruses. Initial infection represents a decisive moment in viral infection deciding the resistance vs. susceptibility, which make observations of the factors driving the initial infection even more relevant.

A large number of studies have used infectious clones; however, effective comparison of the disease incidences between the studies is difficult as ultimately variations in cultivar and virus strains are the main determinants, which are often not the focus of the investigations. In addition, many factors, such as the age of the plant, light and environmental conditions, are often unrecorded and unlikely to follow a prevalent standard. However, a few experiments have optimised various parameters, such as density of agrobacterium or age of plant, and reviewing this limited evidence gives us guidelines to optimise conditions for new experiments. Obtaining high rates of successful infections from agrobacterium or biolistic inoculations will give high levels of confidence in the data we generate, which is crucial for subsequent research.

## Whitefly transmission

Different members of *Geminiviridae* are spread by different insect vectors (Wang and Blanc 2021). Within the begomovirus group, different species of whiteflies can have preference for specific begomoviruses due to their feeding preference for certain host plants and the subsequent co-adaptation (Maruthi et al. 2002; Campbell et al. 2023). The geminivirus-transmitting insects all feed on plants by piercing the leaf with their mouthpiece and sucking the sap from the vascular system and either acquiring or delivering the virus in so doing. Insect transmission depends on several factors including the amount of virus carried by the insect and the amount of feeding (Wang et al. 2022a; Janssen et al. 2022). A single whitefly fully loaded on begomovirus can deposit viral DNA, into the leaf, in the range of nanograms (Roy et al. 2021). Comparing this to agrobacterium or mechanical inoculation may not be meaningful as there is no accounting for the amount of viral DNA which makes it into cell nuclei by these methods. In insect transmission, the CP not only assists with entry of the single-stranded DNA (Krichevsky et al. 2006) but can also have functions that are critical for the establishment of infection (Vo et al. 2023). The insect vectors are not only obedient carriers of the virus as they feed on plants, but also deliver effectors to the plant that assist the insect in feeding whilst also modifying the plant immune signalling. In return, the virus can make plants easier to feed on (Naalden et al. 2021; Ray and Casteel 2022). Whiteflies therefore represent the most certain way of geminivirus transmission. The malvastrum leaf curl Guangdong virus (MLCuGdV), for example, was unable to initiate infection in its natural host *Malvastrum coromandelianum* via agroinfection, whilst whiteflies were able to transmit (Wu et al. 2007; Guo et al. 2008). However, whitefly colonies are difficult to maintain, and they are not allowed to be maintained in some countries due to quarantine reasons. Developing virus infectious clones therefore is the easier way of researching geminiviruses on aspects that do not require transmission by the insect vectors.

## The plant immunity

*N. benthamiana* is commonly used as the first host for testing virus infectivity owing to its susceptibility to plant viruses, which is due to deficiencies in its silencing pathways (Yang et al. 2004; Goodin et al. 2008; Fujiuchi et al. 2016; Bally et al. 2018). It also helps that it is easy to fill the apoplastic space of *N. benthamiana* with agrobacterium suspension which allows it to reach the phloem area (Guan and Zhou 2006). In most other plants, leaf infiltration is far less efficient due to their rigid leaf morphologies. Although it is not a limitation on agrobacterium to deliver the DNA in the wounded area, it is the various mechanisms of gene silencing, which prevents initial expression and thereby virus infection (Wroblewski et al. 2005; Dunoyer et al. 2006; Bilichak et al. 2014; Zhang et al. 2020; Azizi-Dargahlou and Pouresmaeil 2023).

Multiple plant immunity pathways silencing the T-DNA carrying virus constructs are reviewed (Vaucheret 2022; Roca Paixao and Déléris 2025). In brief, once ‘detected’ small double-stranded RNAs (dsRNAs) are produced from the invading DNA. These are converted by DICER ribonucleases into small interfering RNAs and loaded onto the RNA-induced silencing complexes which targets the corresponding DNA sequence for methylation and the formation of heterochromatin preventing virus protein expression, or targeting the expressed viral mRNAs for cleavage, removing them. That is, of course canonical and simplified, as viruses have ways of countering the host silencing which has evolved secondary pathways to circumvent it (Gupta et al. 2021; Zhang et al. 2023). What is noteworthy here regarding the initial geminivirus infection is the systemic spread of the RNA interference (RNAi) silencing response through systemic movement of siRNA in the plant (Molnar et al. 2010; Qin et al. 2012; Zhang et al. 2019; Sanan-Mishra et al. 2021). The activation of RNAi negatively affects geminivirus infectious clone (Kumar et al. 2017; Sangwan et al. 2023). This could contribute to an activation of siRNA mechanism in wider leaf area as it would occur during leaf infiltrations. Having a large number of cells with active siRNA could lead to the overall abortion of virus infection. Therefore, leaf infiltration of agrobacterium is seemingly less effective than, for example, stem injections where the virus is potentially introduced into phloem tissues, which are the preferred by geminiviruses.

## The mechanism of DNA transfer in agroinfections

**Fig. 2:**
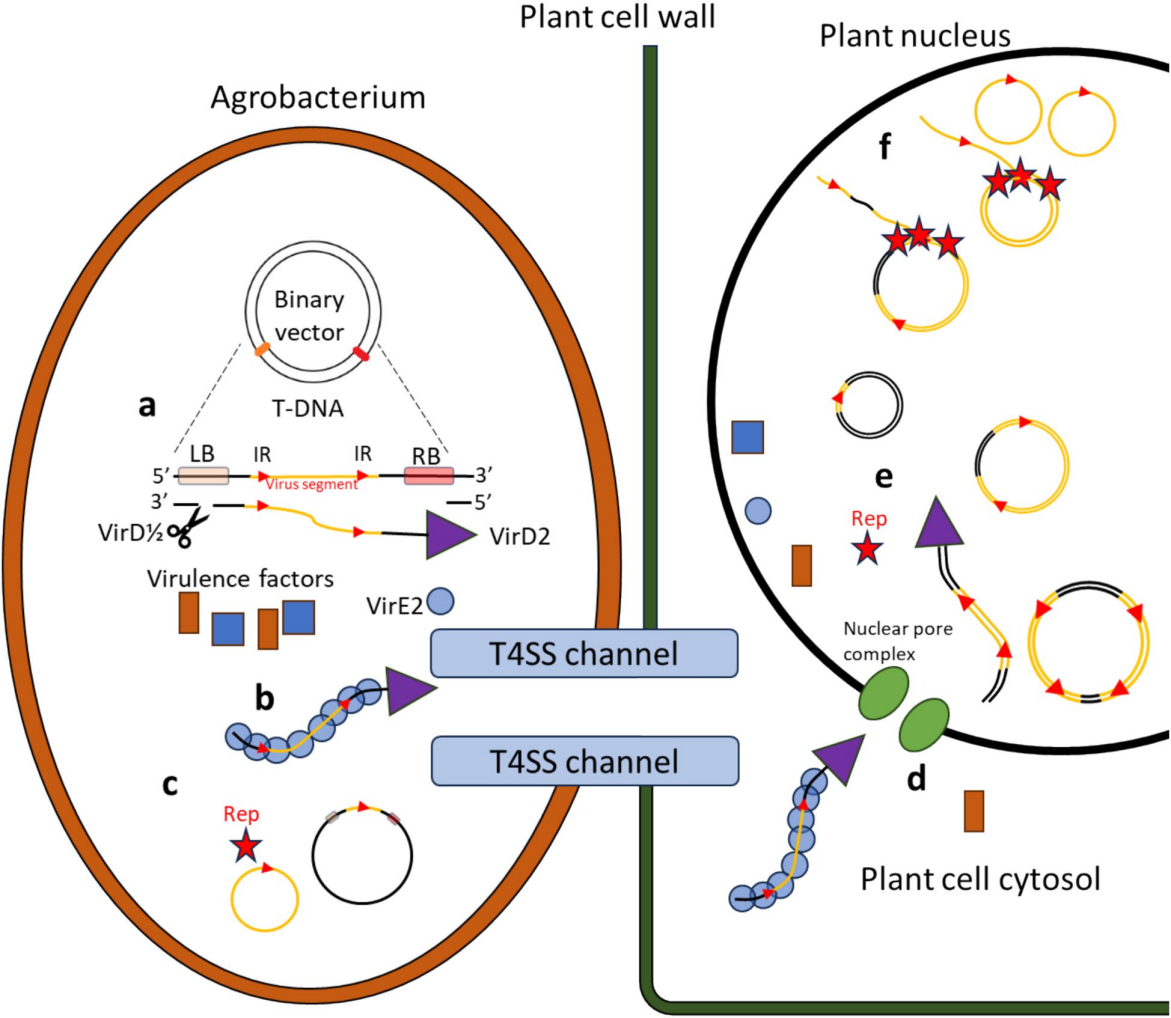
Chart describing the journey of geminiviral DNA from agrobacterium binary vector to circular DNA in the plant cell nucleus. **a.** VirD2 in conjunction with VirD1 nicks DNA, VirD2 binds to the right border (RB) of the T-DNA, excising a single-stranded DNA from the binary T-DNA vector. The DNA contains an infectious dimer of a geminivirus. **b.** The nascent T-DNA is coated with VirE2 and then transferred through the T-pilus T4SS channel into the plant cell cytosol along with other virulence factors. **c.** Regardless of the two previous steps residual expression of Rep by the agrobacterium allows circular DNAs of the virus to form within the agrobacterium. This circular DNA plays no part in infection (Grimsley et al. 1987). **d.** VirD2 and VirE2 interact with the nuclear pore complex to import the T-DNA to the plant cell nucleus. **e.** The T-DNA is subjected to the plant DNA repair mechanisms turning the single-stranded DNA into various double-stranded forms and the Rep is expressed. **f.** The Rep will initiate replication and liberate single circular virus genome virus from the T-DNA, the order of which is unclear

For the infectious clones, the viral DNA is delivered as the dimer and is then eventually converted to a circular DNA, which makes the details of agrobacterium DNA delivery worth considering. As nature’s natural genetic engineer, the bacterium inserts a transfer-DNA (T-DNA), held in its tumour inducing plasmid (Ti-plasmid), encoding genes which when inserted into the plant genome induces tumour formation and engineer the plant to produce the opines only agrobacterium can metabolise. The Ti-plasmid are about a quarter million base pairs large and, in addition to the T-DNA, also contain many of the virulence genes involved in T-DNA delivery. Although, most agrobacterium strains used for plant transformation have had their tumour inducing native T-DNA removed. As the agrobacterium T-DNA is selected for transfer by a short left and right border sequence, the binary plasmids can transfer the geminivirus as T-DNA, by inserting it between the border sequences. One sidenote of geminivirus DNA in agrobacterium is that agrobacterium will also express the Rep protein which then creates circular DNAs inside the agrobacterium by its nicking and re-ligation activity (Selth et al. 2002) (Fig. 2c). Agrobacterium delivers multiple copies of T-DNA through multiple copies of the binary plasmid (Oltmanns et al. 2010) and thus deliver multiple copies of viruses into a plant cell to initiate virus infection. The agrobacterium enzyme VirD2 nicks the Ti-plasmid DNA at the right T-DNA border and covalently binding itself to the 5’-end pulling out a single-stranded DNA (Jasper et al. 1994). Agrobacterium coats the T-DNA with the VirE2 protein which protects it on its way to the plant nucleus (Fig. 2b) (Christie 2004; Li et al. 2020). The agrobacterium essentially creates a channel, the T-pilus into the plant cell to transfer both the DNA and agrobacterium virulence proteins (Nester 2015). Whilst discussion is ongoing as to the exact mechanisms of T-DNA integration into the plant genome (Gelvin 2021; Thomson et al. 2024), integration is not needed as part of the transient expression, like for geminiviruses (Singer 2018). The single-stranded DNA is converted to double-stranded DNA with some ending up as various circular “T-Circle” T-DNAs. Often two T-DNAs will fuse tail–tail first, as VirD2 remains covalently bound, before becoming circular through a recombination process (Fig. 2e) (Singer et al. 2012; Gelvin et al. 2022). Getting the geminiviral T-DNA dimer into the nucleus is only one step, to become a virus infection, the geminivirus must be ejected from the T-DNA (Fig. 2f). The ejection of the circular viral DNA from the dimer is, like the replication mechanism, not fully understood, as these could also escape through recombination-dependant means (Bonnamy et al. 2023). In mechanical inoculation, viral DNA also retains infectivity as linear DNA, because the host repair mechanisms readily re-ligates it to a circular molecule (Lapidot et al. 2007). Inoculation with plasmid DNA containing a single copy of the virus should not be infectious as the plasmid backbone interrupts the virus sequence. However, monomeric clones of pepper huasteco yellow vein virus was infectious as a plasmid if the plasmid backbone was inserted into the coat protein, with the resulting systemic virus infection having reverted to the natural size. The coat protein is not necessary for its replication and thus the entire plasmid must undergo replication, with recombination eventually ejecting the intervening plasmid sequence (Bonilla-Ramírez et al. 1997; Lapidot et al. 2007). This is particularly true in geminiviruses as they appear to be selective towards their native size (Bisaro 1994). The recent discovery of superinfection exclusion mechanism complicates the matter somewhat because it is possible to speculate on some mechanisms where only a few initial copies enter rolling circle replication (Ren et al. 2023), e.g. Reps nicking and joining activity for the release of circular DNA is inhibited by a plant protein, proliferative cell nuclear antigen (Bagewadi et al. 2004). If both rolling circle replication and recombinant-dependant replication occur, it is possible that the balance is shifted as comparison of DNA forms between systemic and infiltrated tissue suggest the recombination-dependant form is emphasised in the initial agroinfection (Jeske et al. 2001). Leading to the question, does agrobacterium inoculation essentially force the virus to escape from a pool of defective viral DNAs, essentially slowing true initiation of infection which in turn gives the plant silencing a leg up?

## Agrobacterium strains

Agrobacterium is a pathogen on its own and in addition to DNA, it also transfers virulence proteins to the plant which disables the bacterial defence response and promotes transformation (Lacroix and Citovsky 2022). Notably, the agrobacterium effector 6b can interfere with the silencing machinery (Wang et al. 2011). Much like geminivirus strains have preferred hosts, so too do the strains of agrobacterium. The individual lab strains can also differ in their chromosomal background, Ti-plasmid and/or small genomic alterations such as loss or gain of a selection marker (Hellens et al. 2000; De Saeger et al. 2021), which makes optimising the methods across different research teams challenging. For example, the C58C1 (pTiB6S3ΔT)^H^ is the C58 chromosomal background with its native Ti-plasmid replaced by the Ti-plasmid from the wild strain B6S3 where the T-DNA region has been deleted (Petit et al. 1978; Deblaere et al. 1985). Finally, the H denotes that the strain contains the helper plasmid pCH32 which increases the expression of some virulence genes (Hamilton 1997). Unfortunately, often names have been simplified in the plant community, such that C58C1(pTiB6S3ΔT)^H^ has been written as C58C1 and GV3101(pMP90) simply as GV3101. Both these plasmids originally denoted agrobacterium strains without a Ti-plasmid and thus lacking many key genes for the transfer of DNA. In this review, we have not renamed agrobacterium strains from what they are referred to in publications.

In agroinfection of tomato and zucchini plants with tomato leaf curl New Delhi virus (ToLCNDV), changing the agrobacterium strain from AGL1, EHA105 or GV3101 to LBA4404 doubled the efficiency of infection (Ruiz et al. 2017). Here, LBA4404 has the Ach5 chromosomal background whilst AGL1, EHA105 and GV3101 belong to the C58 chromosomal background. Another study with agroinfection of tomato with tomato yellow leaf curl Kanchanaburi virus (TYLCKaV) with two different strains of agrobacterium, GV2260 and EHA105, resulted in different disease incidences (Koeda et al. 2017). The Ach5 background also had higher disease incidence over C58C1 for mungbean yellow mosaic India virus (MYMIV) in mungbeans. Inoculation of DNA-A with Ach5 and DNA-B with C58C1 produced high disease incidence (Table 1) (Jacob et al. 2003), indicating an effect from the agrobacterium effectors. Whilst studies indicate an effect from the choice of Agro strains on different plant species, there is no evidence to select a universally better strain. This calls for more agroinoculation studies comparing the efficiency of different strains whilst keeping all other parameters constant. This is also the case for plant transformations where different plant species are preferentially transformed with different agrobacterium strains (Chetty et al. 2013; Kassahun et al. 2021). Whilst other parallel parameters such as silencing and necrotic response to agroinfections are the major limitations for the expression of the T-DNA by the agrobacterium (Kuta and Tripathi 2005; Zipfel et al. 2006). In *Nicotiana tabacum*, the GV3101 agrobacterium strain induces a stronger defence response than LBA4404, as measured by reactive oxygen species and resistance to *Pseudomonas syringae* (Sheikh et al. 2014). This induction of plant defences inhibited tobacco mosaic virus (TMV) infection in leaf regions previously infiltrated with agrobacterium (Pruss et al. 2008). Comparison of different agrobacterium strains for the stable transformation of *N. tabacum* was likewise better with LBA4404 (Bakhsh et al. 2014).

**Table 1:** Different agroinfection experiments mentioned in this review

Virus (Genbank accession)	Plant—variant	Inoculation method	Agrobacterium strain	Inoculation media	Stage of plant	Infectivity	Reference
Malvastrum leaf curl Guangdong virus (AM236779)	*Petunia hybrida*	Stem puncture	EHA105	Bacterial media	4 to 6 leaf stage	100% (30)	(Wu et al. 2007)
	*Malvastrum coromandelianum*					0% (?)	
Bean golden yellow mosaic virus (AF173555 and AF173556)	*Phaseolus vulgaris*—susceptible cultivars	Stem puncture—first internode	LBA4404	2 × concentration of yeast tryptone media, OD_600_ of 1	10 to 12 days old plants	73% (244)	(Garrido-Ramirez et al. 2000)
	*Phaseolus vulgaris*—resistant cultivars				10 to 12 days old plants	6% (112)	
Tomato yellow leaf curl virus (AJ519441)	*Solanum lycopersicum*—cv. Marmande	Stem puncture	LBA4404	Lysogeny broth, OD likely high	Five to six leaf stage	100% (10)	(Morilla et al. 2005)
	*Capsicum annuum*—cv. Cadia					0% (40)	
Vernonia yellow vein virus (AM182232)	*Vernonia cinerea*	Stem puncture—first internode	EHA105	0.9% NaCl, OD_600_ of 1.2 to 1.5	40 to 45 days old plants	64% (20)	(Packialakshmi and Usha 2011)
		Leaf infiltration			40 to 45 days old plants	0% (?)	
Cucurbit leaf crumple virus (NC_002984 and NC_002985)	*Citrullus lanatus*—multiple cultivars	Stem puncture—beneath the shoot apex and at first internode	C58	Bacterial media, OD_600_ of 1	2 true-leaf stage	77%-87%	(Hagen et al. 2008)
	*Cucurbita pepo*—multiple cultivars					13%-84%	
	*Cucumis melo*—multiple cultivars					0–60%	
Cucurbit leaf crumple virus (PP617367 and PP617368)	*Cucurbita pepo. cv.* Gold Star	Leaf puncture by dermal microneedle roller	EHA105	MM, OD_600_ of 1	2 true-leaf stage	90% (20)	(Kavalappara et al. 2024)
				Water, OD_600_ of 1		100% (20)	
Mungbean yellow mosaic India virus (KY556680 and KY556679	*Vigna unguiculata*—cv. Walp	Leaf infiltration into young trifoliate leaves	EHA105	MM, 200 µM acetosyringone, pH 5.6 OD_600_ of 0.6	4 weeks old	100% (5)	(Kumar et al. 2017)
	*Vigna unguiculata*—cv. Walp. Stable RNAi lines					24% (45)	
Mungbean yellow mosaic geminivirus (AY049772 and AY049771)	*Vigna mungo*—cv. Hepper	Stem puncture (Near cotyledon)	C58 (TiC58)	Agrobacterium resuspended in water	2 days old seedling	40% (30)	(Mandal et al. 1997)
		Seed puncture			Seeds submerged 1 day in water	13% (30)	
Sweet potato leaf curl virus (JX286654.1)	*Nicotiana benthamiana*	Stem puncture	GV3101	MM, 200 µM acetosyringone OD_600_ of 1, pH 5.6	2 weeks old	80% (10)	(Zhang et al. 2024)
				1 day pre-incubation in AB with 200 µM acetosyringone, followed by inoculation in		100% (10)	
				MM with 200 µM acetosyringone, OD_600_ of 1, pH 5.6			
Pepper yellow leaf curl Indonesia virus (LC051114 and AB213599)	*Capsicum annuum*—cv. No. 218	Leaf infiltration on the abaxial sides of the cotyledons	GV2260	MM, 400 µM acetosyringone OD_600_ of 0.1	2 weeks old seedling	92% (13)	(Koeda et al. 2018)
				MM, 400 µM acetosyringone OD_600_ of 1		8% (13)	
Maize streak virus	*Zea mays*	Stem puncture into stem and bundled sheaths	EHA105	MS, pH 5.5	40 to 45 days old plants	88% (43)	(Grimsley et al. 1987)
Mungbean yellow mosaic India virus	*Vigna radiata*—cv. R. Wilczek	Stem puncture	EHA105	Bacterial media, OD_600_ of 1	2 to 3 true leaves stage	98% (132)	(Li et al. 2015)
Mungbean yellow mosaic India virus—point mutation removing AC5 reading frame						24% (132)	
Maize streak virus	*Zea mays*	Stem puncture into stem and bundled sheaths	EHA105	MS, pH 5.5	40 to 45 days old plants	88% (43)	(Grimsley et al. 1987)
Mungbean yellow mosaic India virus	*Vigna radiata*—cv. R. Wilczek	Stem puncture	EHA105	Bacterial media, OD_600_ of 1	2 to 3 true leaves stage	98% (132)	(Li et al. 2015)
Mungbean yellow mosaic India virus—point mutation removing AC5 reading frame						24% (132)	
Tomato golden mosaic virus (NC_001507 and NC_001508) (single construct/agrobacterium)	*Nicotiana benthamiana*	Stem puncture at stem base and 5 cm above	PC2669 (pTiC58)	Yeast extract beef broth, 2 × 10^9^ cells pr. 20 µl	4 to 6 leaf stage	95% (20)	(Hayes et al. 1988)
Tomato golden mosaic virus (NC_001507 and NC_001508) (separate constructs/agrobacterium)					4 to 6 leaf stage	70% (20)	
				Yeast extract beef broth, 2 × 10^4^ cells pr. 20 µl	4 to 6 leaf stage	40% (20)	
				Yeast extract beef broth, 2 × 10^3^ cells pr. 20 µl	4 to 6 leaf stage	0% (20)	
Tomato yellow leaf curl virus (AM282874)	*Solanum lycopersicum*—cv. moneymaker	Stem puncture	EHA105	MS, 100 µM acetosyringone, OD_600_ of 1	4 to 6 leaf stage	48% (?)	(Liu et al. 2021)
		Leaf infiltration				40% (?)	
		INABS				72% (?)	
Tomato yellow leaf curl Kanchanaburi virus (AB921568 and LC177332)	*Solanum lycopersicum*—cv. Momotaro	6 cm of shoots are submerged in agrobacterium suspension	GV2260	MM, 200 µM acetosyringone, pH 5.6, OD_600_ of 0.6	First true-leaf stage	4% (57)	(Koeda et al. 2017)
			EHA105			41% (45)	
		Stem puncture with toothpick coated in agrobacterium	GV2260	Raw agrobacterium		4% (73)	
			EHA105			92% (76)	
Tomato yellow leaf curl virus (AB116632)	*Solanum lycopersicum*—cv. Shugyoku	6 cm of shoots are submerged in agrobacterium suspension	C58C1	MM, 200 µM acetosyringone, pH 5.6, OD_600_ of 0.6	Fifth adult leaf fully expanded	93% (16)	(Yamaguchi et al. 2013)
		6 cm of shoots are submerged in agrobacterium suspension and vacuum infiltrated				100% (16)	
Ageratum yellow vein virus + β-satellite (X74516 and AJ252072) (separate constructs/agrobacterium)	*Ageratum conyzoides*	Stem puncture	GV3850	Water, not detailed	3 to 5 leaf stage	6.3% (63)	(Saunders et al. 2001)
Ageratum yellow vein virus + β-satellite (X74516 and AJ252072) (single construct/agrobacterium)						86% (22)	
Tomato leaf curl New Delhi virus (KF891468 and KF891467)	*Cucurbita pepo*—cv. Victoria or *Solanum lycopersicum*—cv. Marmande	Leaf infiltration, cotyledons and first leaves. Stem puncture	EHA105 or GV3101	Not detailed	First leaves	50% (?)	(Ruiz et al. 2017)
			LBA4404			100% (?)	
Mungbean yellow mosaic virus (AJ132575 and AJ132574)	*Vigna mungo*—cv. Hepper	Stem puncture at hypocotyl and submersion 12 in agrobacterium suspension in darkness	GV2260—co-inoculation with 2 agro strains	AB, 100 µM acetosyringone, pH 5.6	12-h germinated seeds	27% (70)	(Jacob et al. 2003)
			GV2260—single strain carrying 2 plasmids	AB, 100 µM acetosyringone, pH 5.6	12-h germinated seeds	69% (88)	
			Ach5—single strain carrying 2 plasmids	AB, 100 µM acetosyringone, pH 5.6	12-h germinated seeds	100% (38)	
Mungbean yellow mosaic India virus (MN020535 and MN020536)	*Vigna radiata*—cv. Maha Gujarat	Stem puncture at upper tender stem	EHA105	Raw agrobacterium	First trifoliate leaf stage	20% (35)	(Sivalingam et al. 2021)
		Stem puncture at shoot tip				7.5% (40)	
		Stem puncture at epicotyl and one cotyledon		LB medium, OD_600_ of 1, variable acetosyringone	Recently sprouted seedlings with just emerging cotyledon	100% (53)	
		Stem puncture at radical				32% (31)	
Cotton leaf curl Kokhran virus (AJ496286)	*Nicotiana benthamiana*	Leaf infiltration	GV3101	MM, 100 µM acetosyringone, pH 5.6 OD_600_ of 1	Not detailed	100% (20)	(Rasool et al. 2016)
	*Nicotiana benthamiana* transgenics expressing ‘G5’ single-stranded DNA binding protein					32% (40)	
Tomato leaf curl New Delhi virus-OM (GU180095 and MK883714)	*Cucumis melo*—cv. Silver Light	Leaf infiltration	C58	MM, 150 μM acetosyringone, OD_600_ of 0.5	Not detailed	91% (12)	(Lee et al. 2020)
Tomato yellow leaf curl virus (pTYLCV-[JU])	*Solanum lycopersicum*—cv. NS16	2 cm of cut microshoots are dipped in agrobacterium suspension 30s and replanted in solid MS media	GV3101	MS, 100 μM acetosyringone, OD_600_ of 0.125	21 days old sterile grown	45% (20)	(Al Abdallat et al. 2010)
				MS, 100 μM acetosyringone, OD_600_ of 0.25		80% (20)	
				MS, 100 μM acetosyringone, OD_600_ of 0.5		70% (20)	
Tomato leaf curl New Delhi virus-OM (GU180095 and MK883714)	*Cucumis melo*—cv. Silver Light	Leaf infiltration	C58	MM, 150 μM acetosyringone, OD_600_ of 0.5	Not detailed	91% (12)	(Lee et al. 2020)
Abutilon mosaic virus (X15983 and X15984)	*Nicotiana benthamiana*	Stem puncture	LBA4404	Not detailed	Not detailed	100% (5)	(Wege and Pohl 2007)
Tomato yellow leaf curl virus (AM282874)	*Solanum lycopersicum*—cv. Pufen7	Leaf infiltration	Not detailed	MM, 200 µM acetosyringone, OD_600_ of 1.5–2	Two true-leaf stage	83% (24)	(Zhang et al. 2021)
					28 days after the two true-leaf stage	46% (24)	
Okra enation leaf curl virus and bhendi yellow vein mosaic betasatellite (KJ462074 and KJ462076)	Abelmoschus esculentus—cv. N-568	Leaf infiltration	EHA105	MM, 150 µM acetosyringone, OD600 of 1.6	4–5 weeks old	0%	(Gupta et al. 2022)
		Petiole puncture				0%	
		Hypocotyl puncture, followed by 5-h incubation in agrobacterium LB media		LB medium 200 µM acetosyringone, OD600 of 1.0	36 h old seedling	0%	
		Shoot apex puncture (injection into the terminal buds)		33% agrobacterium/MM buffer as v/v, 250 µM acetosyringone	7–8 leaf stage	50% (120)	

## Inoculation designs and optimisation

It is not just the agrobacterium strain which influences geminivirus infection, the where and when of the agroinoculation also influence the disease incidence. The relative expression of relevant plant genes for infection and defence or lack thereof undoubtedly varies in place and time in the plant. Summaries of studies on these topics are listed in Table 1. Various methods have been tried such as the injection of agrobacterial suspension on the abaxial surface of the leaf with water, buffer or nutrient media, stem injections and/or inoculation of the growing tips amongst other methods.

In some of the first attempts to initiate viral infection, the agrobacterium resuspended in yeast extract broth (YEB) media was injected into plant stems with a syringe (Hayes et al. 1988). In *N. benthamiana*, agrobacterium suspension is pressed into the abaxial side of leaves with a needleless syringe, which distributes the bacteria on a wider area to facilitate easier virus infection. Stem puncture/injection was also tried which is often successful at initiating infections even when agrobacterium is resuspended in its liquid growth media. However, rich liquid media is a poor inducer of virulence genes, and most transient expression experiments use specialised buffers (Krenek et al. 2015). An additional step where agrobacterium was induced overnight in a virulence enhancing buffer resulted in higher virus titres and more severe symptoms for sweet potato leaf curl virus (SPLCV) agroinfection of *N. benthamiana* (Zhang et al. 2024). On the other hand, using water instead of the usual MgCl/MES buffer as inoculum gave more severe infections when pricking leaves of zucchini (Kavalappara et al. 2024). The composition of the infiltration buffer has markedly influenced the transient expression efficiency. The pH of the media can also influence virus infection; for example, a pH 5.5 infiltration buffer interfered with responses of *Arabidopsis* to agrobacterium through pathogen-associated molecular pattern (Ranf et al. 2011; Wang et al. 2018; Wu and Lai 2022). Of the studies listed here (Table 1), many do not use the supposedly optimum magnesium MES buffer for agroinfection with the rest often using the growth media, and there is thus a lack of reported evidence towards this being crucial. In one of the earliest reports of agroinfection experiments, disease incidence remained unchanged for different concentrations of agrobacterium by stem pricking of *N. benthamiana*, unless less than 10,000 agrobacterium cells were used, which is below any normal OD_600_ (Hayes et al. 1988). In the tomato top stem injection method, an OD_600_ of 1.0 was claimed to be optimal, with the same being the case for mungbeans and MYMIV (Liu et al. 2021; Sivalingam et al. 2021), although differences were minor and only the 0.5–1.5 range was tested. In contrast, infiltration of pepper cotyledons with dilutions of agrobacterium had significantly increased disease incidence at 0.1 OD_600_ compared to 1 (Table 1) (Koeda et al. 2018), whilst another stem cutting infiltration protocol on tomato had the highest disease incidence of 80% at 0.25 OD_600_ compared to 45% at 0.125 OD_600_ or 70% at 0.5 OD_600_ (Al Abdallat et al. 2010). Generally, an OD_600_ of 0.5 to 1 is used as that tends to be considered optimal for most transient expression experiments. However, an OD_600_ of 0.2 was found to be optimal for high transient expression of geminiviral replicons in some studies (Leuzinger et al. 2013). Higher concentrations of agrobacterium stress the plant more, without improving the viral DNA delivery to initiate infection.

**Fig. 3:**
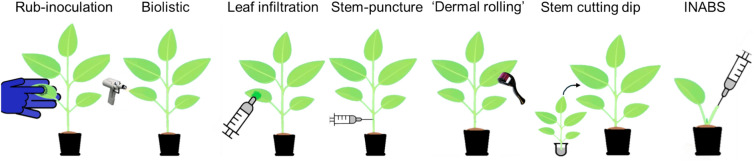
Schematic presentation of different inoculation methods. Rub-inoculation with abrasive and plasmid–DNA, virus–DNA or virus particles. Biolistic with DNA covered particles. Leaf infiltration with an agrobacterium suspension. Stem puncture where a needled syringe injects the agrobacterium into the stem. Dermal rolling, whereby the leaves are prick inoculated with a dermal roller, stem cutting dip where cuttings young stem cuttings are dipped in agrobacterium solution before being replanted. INABS, whereby a cutting is injected with agrobacterium suspension into its cut stem before being replanted and the adventitious shoot is grown as a new main plant

Exactly where on the plant the agroinfection takes place is another factor which impacts disease incidence. Agroinfection with MYMIV was significantly more effective when delivered to or near the epicotyl region of bean sprouts, indicating that inoculating younger parts of the plants can give a higher success rate. Older seedlings had lower disease incidence and injections into their tender stems gave a higher disease incidence than the shoot tip (Mandal et al. 1997; Sivalingam et al. 2021). For tomato or sweet potato, which can be propagated from cuttings, one inoculation approach took seedlings and cut them at the stem, dipping them in an agrobacterium suspension before repotting. Disease incidence was not significantly improved by vacuum infiltration and lengthier soaks for tomato seedlings inoculated with tomato yellow leaf curl virus TYLCV (Yamaguchi et al. 2013). Such an approach was claimed to allow symptomless infection by TYLCV of an otherwise resistant tomato cultivar (Al Abdallat et al. 2010). Although without a comparison to stem or leaf using the same virus cultivar combination, it is not possible to properly assess if there were any improvements to disease incidence. Later, the approach was compared to pricking the stem with solid agrobacterium and found less effective (Koeda et al. 2017). Cutting the apical bud of tomato and injecting agrobacterium into the upper stem, dubbed INABS (Fig. 3), had a higher disease incidence than leaf infiltration or stem base injection, which had comparable efficiency in the experiment (Liu et al. 2021), and for the vernonia yellow vein virus (VeYVV) in vernonia, leaf infiltration was claimed to be ineffective whilst stem injecting had a disease incidence of 64% (Table 1) (Packialakshmi and Usha 2011). Okra enation leaf curl virus (OELCuV) and bhendi yellow vein mosaic betasatellite (BYVMB) were only successful agroinfection when a high concentration of agrobacterium was injected into the terminal buds of the shoot apex, whereas leaf infiltration and injection into petioles or seedlings were unsuccessful (Gupta et al. 2022). Another alternative approach for inoculation is to use a dermal microneedle roller to rapidly apply agrobacterium suspensions to leaves, attempting to mimic the whitefly feeding; the method was reported to give symptoms similar to whitefly-based inoculation in squash (Kavalappara et al. 2024). It would thus appear that plant stress responses to wounding increase susceptibility to geminivirus infections. Exact plant age and health also influence infection rate generally as young seedlings are considered more susceptible to virus than older mature plants (Fargette et al. 1993; Zhang et al. 2021). In one experiment, disease incidence dropped from around 70% to 10% if the pepper seedlings were grown for just one extra week (Koeda et al. 2018). Environmental factors have the potential to break resistance, as is the case for TYLCV, wherein resistant cultivars of tomato become susceptible at higher temperature (Koeda and Kitawaki 2024). Effects are dependent on plant–virus combinations; in cassava, several viruses had reduced DNA load at 30 °C compared to 25 °C (Chellappan et al. 2005; Velásquez et al. 2018). At least within a single experimental setup, the environmental factors should be kept constant by design.

Given many begomoviruses require the dimers of DNA-A and DNA-B to be co-delivered, normally done by mixing different agrobacterium cultures containing the DNA-A and DNA-B containing plasmid, it was considered if including dimers of both on the same plasmid could increase disease incidence if delivery is the limiting factor. This was tested for tomato golden mosaic virus (TGMV), and it achieved slightly higher disease incidence in *N. benthamiana*, 19/20 compared to 14/20. Meanwhile, disease incidence for DNA-A transgenic *N. benthamiana* inoculated with DNA-B was low at 2/20 (Hayes et al. 1988). Having both individual dimers of DNA-A and DNA-beta in a single T-DNA increased the disease incidence for ageratum yellow vein virus (AYVV) in ageratum from 4/63 to 19/22 for ageratum, although disease incidence remained 100% for *N. benthamiana* (Saunders et al. 2001). Instead of having both DNA-A and DNA-B in one dimer, it is possible for a single agrobacterium to contain and launch multiple distinct T-DNAs. By integrating the geminiviral dimer into the agrobacterium Ti-plasmid, it was possible for one agrobacterium to carry both DNA-A and DNA-B dimers as separate plasmids and T-DNAs. This approach increased disease incidence of MYMIV in mungbeans from 27 to 69% (Table 1) (Jacob et al. 2003). In *N. benthamiana*, the multiplicity of agroinfection has been estimated, and an OD_600_ of 0.2 of each agrobacterium used in co-inoculation is sufficient to ensure virtually every affected cell received T-DNA from all agrobacterium used (Carlson et al. 2023). Faba bean was agroinfected with eight separate genomic components of a nanovirus (family *Nanoviridae*) co-delivered to enable infection (Timchenko et al. 2006). These results are indicating that co-delivery is not a limiting factor in the virus infection processes. Potentially, some observations regarding single construct vs. co-inoculation might be explained by the presence of a preferred ratio of DNA-A/DNA-B in an individual plant cell (Xiao et al. 2023). It is suggested that whiteflies somehow preferentially acquire DNA-A and DNA-B of ACMV in a 1:1 ratio (Kennedy et al. 2023), although the mechanisms require a thorough investigation.

## Mechanical and biolistic method of virus delivery

Whilst agrobacterium is the main artificial inoculation method, mechanical and biolistic inoculation are also worth mentioning. Mechanical transmission, whereby infected plant sap is rubbed onto the leaf, usually with an abrasive to cause local lesions, damages the leaf enough to allow the virions to enter cells. This would be the simplest way of transmission, but only a few geminiviruses can be transmitted to select host plants this way (Morales et al. 1990; Rojas et al. 2005; Lee et al. 2020). For some virus–host combinations, mechanical inoculation is possible whilst only using naked plasmid DNA. It is possible for plasmid DNA to enter the nucleus unaided, even if it is highly inefficient (Bai et al. 2017). Comparable optimisation is limited to the DNA concentrations and buffers used, as the extent of wounding lacks a measure. When rub-inoculating with higher concentrations of DNA, up to 0.4 µg/µl, the disease incidence of TGMV in *N. benthamiana* was increased (Table 2) (Ascencio-Ibañez and Settlage 2007).

**Table 2:** Different mechanical inoculation experiments mentioned in this review

Virus (Genbank accession)	Plant—variant	Inoculation method	Stage of plant	Disease incidence	References
Bean dwarf mosaic virus (unknown)	*Phaseolus vulgaris—*cv. Topcrop	Sap rub inoculated with carborundum dusting (600 mesh) into leaves using infected leaves ground in 1:4 w/v 0.1 M KPO_4_	8 days old	100% (?)	(Morales et al. 1990)
	*Phaseolus vulgaris—*(different cultivars)			0–100%	
Tomato leaf curl New Delhi virus-OM (GU180095 and MK883714)	*Cucumis melo*—cv. Silver Light	Sap rub inoculated with carborundum dusting (600 mesh) into leaves using N. benthamiana infected leaves ground in 1:20 w/v 0.01 M KPO_4_	Cotyledon explants	42% (26)	(Lee et al. 2020)
Tomato golden mosaic virus (K02029)	*Nicotiana benthamiana*	Rub inoculated, carborundum-dusted leaves	Cotyledon explants	66% (6)	(Ascencio-Ibañez and Settlage 2007)
		20 µl 0.03 µg/µl plasmid DNA in water			
		Rub inoculated with carborundum dusting into leaves using 20 µl 0.4 µg/µl plasmid DNA in water	Cotyledon explants	100% (10)	
Indian cassava mosaic virus (AY730035 and AY730036)	*Nicotiana benthamiana*	Rub inoculated with carborundum dusting into leaves using 20 µl 0.1 µg/µl circular viral DNA	Five leaf stage	21% (101)	(Rothenstein et al. 2005)
Indian cassava mosaic virus (AY730035 and AY730036)	*Nicotiana tabacum*—cv. Samsun nn			0% (?)	
		Biolistic using gold particles (1 μm) loaded with 1 µg circular viral DNA using 900 to 1100 psi acceleration		20% (15)	
Abutilon mosaic virus (X15983 and X15984)	*Nicotiana benthamiana*	Rub inoculated with carborundum dusting into leaves using sap derived directly from infected *Nicotiana benthamiana* leaves	Three to five expanded leaves	5% (64)	(Wege and Pohl 2007)
	*Nicotiana benthamiana* (DNA-B transgenic)			31% (35)	
Sri Lankan cassava mosaic virus (KC424490)	*N. benthamiana*	Biolistic using gold particles loaded with 1 μg of RCA product using 200 psi acceleration	30 days old	85% (66)	(Kushawaha et al. 2015)
		Biolistic using gold particles loaded with 1 μg of RCA product using 150 psi acceleration		33% (15)	
		Biolistic using gold particles loaded with 0.7 μg of RCA product using 150 psi acceleration		22% (36)	
Bean dwarf mosaic virus (M88179 and M88180)	*Phaseolus*	Biolistic, gold particles loaded with 2.5 μg of linearised infectious dimer using a 3.1 MPa	3-day-old hypocotyls, grown sterile in the dark	80% (25)	(Levy and Czosnek 2003)
	*Vulgaris*—cv. Benton				
Tomato yellow leaf curl virus (X15656)	*Solanum lycopersicum*	Biolistic using tungsten particles loaded with 5 μg of total DNA from infected plants using 800 or 1200 psi acceleration	Four leaf stage	0% (?)	(Ramos et al. 2003)
Tomato mottle Taino virus (AF012300 and AF012301)				100% (?)	
Tomato yellow leaf curl virus (AJ519441)	*Solanum lycopersicum*—cv. Marmande	Biolistic using gold particles (1 μm) loaded with circular dimer plasmid DNA using 650 psi acceleration	Five to six leaf stage	57% (7)	(Morilla et al. 2005)
	*Capsicum annuum*—cv. Cadia			50% (4)	
Bean golden yellow mosaic virus (AF173555 and AF173556)	*Phaseolus vulgaris*—susceptible cultivars	Biolistic, gold particles loaded with 0.83 μg or 0.42 μg of circular dimer plasmids using 1500 psi acceleration	2-day-old seedlings	88% (29)	(Garrido-Ramirez et al. 2000)
	*Phaseolus vulgaris*—resistant cultivars			40% (77)	
Bean golden yellow mosaic virus (AF173555 and AF173556)	*Phaseolus vulgaris*—susceptible cultivars	Sap rub inoculated with celite dusting into half of the expanded primary leaves using infected bean leaves ground in 1:5 w/v 0.1 M KPO_4_	Ten-day-old seedlings	75% (61)	
	*Phaseolus vulgaris*—resistant cultivars			41% (112)	

Biolistic DNA delivery/particle bombardment, where the DNA is coated onto gold or tungsten nanoparticles which are ‘shot’ into the epidermal layer of the leaf, is more effective than the rub inoculation as DNA is delivered into the cell nucleus (Levy and Czosnek 2003; Altpeter et al. 2005). The Indian cassava mosaic virus (ICMV) can be rub inoculated onto *N. benthamiana* but not to *N. tabacum*. Yet, ICMV can be transmitted to *N. tabacum* with biolistic inoculation (Rothenstein et al. 2005), showing the superiority of biolistic inoculations. Likewise, TYLCV cannot be mechanically transmitted but reaches 37% disease incidence on biolistic-inoculated tomatoes (Table 2). Mechanical non-transmissibility is not solely a result of being phloem limited, as there are geminiviruses, such as maize streak virus which can be spread outside the vascular tissue but cannot be mechanically transmitted (Lucy et al. 1996; Wege and Pohl 2007). Virus inoculation optimisation parameters therefore include the particle type, tungsten or gold, particle size, pressure/velocity and distance between gene gun and leaf to be inoculated. In general, optimisation of transient expression by biolistic inoculation depends heavily on the chosen tissue and plant target, making it difficult to compare optimised parameters for geminivirus inoculation. The Sri Lankan cassava mosaic virus (SLCMV) had higher disease incidence with biolistic delivery with higher pressure/velocity (84% at 200 psi vs. 33% at 150 psi) in *N. benthamiana* (Kushawaha et al. 2015). This of course depends on the age and hardiness of the leaf being inoculated with older mature leaves requiring higher pressure than the younger leaves. There is also a trade-off between delivering more and damaging the plant tissue too much in the process (Ueki et al. 2009; Hamada et al. 2017; Lacroix and Citovsky 2020). A standard universal method cannot therefore be recommended for all virus and plant species; a method should always be trialled if 100% infection (or close to) is not achieved on susceptible varieties. Naked DNA rub inoculated onto a plant may not come with the agrobacterium factors, but plants can detect the presence of external DNA which has been wetted onto the plant. They even appear to have different responses depending on if the external DNA is obtained from the plant or if it is from a heterologous organism, with nonself-DNA triggering the hypersensitive response and initiating a systemic acquired resistance (Bhat and Ryu 2016; Chiusano et al. 2021). The mechanism for this nonself-DNA detection is largely unknown (Lozano-Durán 2024). Comparisons between agroinfection and biolistic delivery are rare with only a limited number of studies using multiple methods. A clone of the tomato yellow leaf curl Sardinia virus (TYLCSV) was transmitted to tomato with agrobacterium but not biolistically (Ramos et al. 2003). In the same experiment, a variant of TYLCV was also not infectious biolistically. However, agroinfection failed to cause disease in pepper, with another variant of TYLCV, whilst biolistic method succeeded with low disease incidence (Morilla et al. 2005), which is a clear demonstration of the effect of differences between plant species on agroinfection (Table 1 and 2). Perhaps a combination of lesser preference for peppers, lower virus titres, combined with basal defence response to agrobacterium gave resistance, which is why a testing of the different methods must always be carried out on known susceptible varieties. At the same time, the virus being phloem limited offers a partial explanation as to why biolistic delivery is less efficient. In a screen of different cultivars of beans for resistance to bean golden yellow mosaic virus (BGYMV), inoculation was carried out with both agrobacterium, sap rub and particle bombardment. In this experiment, agroinfection gave lower disease incidence in resistant cultivars than particle bombardment or sap rub (Garrido-Ramirez et al. 2000) indicating that one approach does not work for all virus–plant combinations and/or that optimisation of approach is more important. Generally, biolistic delivery is less common as it is more laborious and costly, requiring preparation of the individual shots for each plant.

## Other considerations regarding geminivirus infectious clones

*N. benthamiana*, owing to the ease by which it can be infected by most geminiviruses, readily provides a host for testing the geminivirus infectivity assays. However, it is resistant to insect feeding and thus difficult to use as a source plant for insect transmission (Davino et al. 2009). Recently, however, gene-edited *N. benthamiana*, made palatable for whiteflies by a deficiency in acylsugar, was found to sustain whiteflies for transmission (Feng et al. 2022; Thompson et al. 2024). Such plants can be used if transmission by insects is an essential part of your studies.

Agroinfection methods have often sought to increase agrobacterium delivery to overcome low disease incidence. Visually tracking the initial infection would be useful in understanding the causes of inoculation escape. However, one of the limitations is the lack of easily detectable markers for virus infections. Geminiviruses select for their genome size (Etessami et al. 1989; Rojas et al. 1998), which makes it impossible to add a marker such as a GFP cassette to the virus coding sequence without compromising its virulence and function in some way. The only exception to this is a few bipartite begomoviruses that retain movement with their CP replaced with GFP, which is unnatural and makes the GFP expression dependent on initiation of viral replication (Padidam et al. 1995; Sudarshana et al. 1998; Levy and Czosnek 2003).

To overcome the limitations of efficient gene expression, *Agrobacterium* strains have been developed with new engineered agrobacterium strains delivering additional effectors to suppress host defences. The reduction in silencing increases the expression and transformation. This has not been tested on geminivirus infection, and whether this would translate into higher disease incidence remains to be seen (Raman et al. 2022). Another upcoming technology to pay attention to is the development of nanoparticles for DNA delivery to plant cells (Cunningham et al. 2018). Plasmid DNA linked to cell penetrating peptides has been shown to enter leaves and express the β-glucuronidase reporter gene (Thagun et al. 2022). Such nanoparticles carrying the viral DNA could be injected into plants like agrobacterium. How and if they affect disease incidence also remain unknown (Zuverza-Mena et al. 2017).

Whilst some virus–host combinations allow an infection to start and spread if any cell is infected, many fail to establish outside of the phloem. The right virus in the right cell can make all the difference for the development of disease. Initial infection by geminivirus has evolved around the assumption that the virus is deposited into the phloem by the feeding insects and not into the mesophyll tissue by humans. Silencing of virus replication by the plant in the mesophyll tissue might be a reason for the failed infections by the geminivirus clones. The silencing machinery also spreads in mesophyll, leading to a potential dynamic where siRNA from aborted infections silences the DNA reaching the phloem before the virus does. A better understanding of the underlying mechanisms in the initial infection would therefore also be connected to a better understanding of tissue tropism of geminiviruses. Comparison between biolistic and agrobacterium-mediated inoculations is rare, as very few studies use both approaches. Such studies would be crucial for uncovering the impact of phloem limitations in initial geminivirus infection. There is a question of whether agrobacterium delivery assists in the generation of defective interfering DNAs, incomplete replicated virus DNAs molecules which may assist in promoting RNAi (Patil and Dasgupta 2006; Bach and Jeske 2014). In addition, stable expression of VirE2 in plants attenuates symptoms and reduces begomovirus DNA accumulation (Sunitha et al. 2011; Resmi et al. 2015; Yousaf et al. 2020), possibly due to the single-stranded binding, as single-stranded DNA binding proteins interfere with begomoviruses (Rasool et al. 2016). Part of the continued research into understanding distinct virus factors found throughout the family *Geminiviridae* involves recombinant viruses (Li et al. 2015; Lee et al. 2020; Vo et al. 2023). A high success rate with infectious clones will aid in easier interpreting of results and reduce the workload and number of plants to be tested.

## Conclusions

Comparison of experimental methods in the literature is challenging considering factors which majorly influence disease incidence might not have been recorded. Exactly where on the stem the plant is pricked, the exact pH of the infiltration buffer, exact conditions of the plant and more can all matter. Plant susceptibility and virus host range can come down to small variations, further complicating like-for-like comparison. It is therefore hard to suggest a universal approach to limit inoculation escape. We therefore provide the following general guidelines: *i*) carry out the experiment in a single setup to limit unknowable environmental variables; *ii*) young seedlings of not more than 2–3 weeks of age are preferable for agroinfection; *iii*) stem puncture is a safer bet than leaf infiltration; *iv*) injecting the epicotyl region or upper stem is better; *v*) changing agrobacterium background and/or Ti-plasmid can significantly alter disease incidence, if the initial trials do not work out; *vi*) optimising agrobacterium virulence gene induction is unlikely to improve disease incidence; *vii*) OD_600_ of 1 is common, but lowering it tenfold might in some cases improve disease incidence; *viii*) for bipartite begomoviruses, unless swapping DNA-B components is part of the plan, it could be advantageous to clone DNA-A and -B into one vector. Finally, with the variability introduced by the methods, it would be advisable to test the system on a known susceptible variety and alter the conditions if high levels of infections (> 100% or close to it) are not achieved consistently.

The infectivity is the percentage of inoculated plants developing symptoms, with the total number of plants tested in the experiment noted in parenthesis, if provided; otherwise, a (?) is shown. Genbank accession of infectious clones is provided if available; otherwise, variant information is provided. For methods, see (Fig. 3). Agrobacterium strains are written without Ti-plasmid if unmentioned in their publication. Bacterial media—the liquid media used to grow the bacterium was used as inoculation; this is likely yeast extract beef broth, yeast extract tryptone or lysogeny broth media, but the publication does not clarify it. *OD*_*600*_ optical density at 600 nm, *cv*. cultivar. Inoculation media abbreviations, *MM* magnesium and MES, common inoculation buffer with 10 mM MgCl_2_, 10 mM MES and pH usually 5.5 to 5.8; *AB* agrobacterium minimal media (Wu and Lai 2022), *MS* Murashige and Skoog basal medium (Murashige and Skoog 1962).

The infectivity is the percentage of inoculated plants developing symptoms, with the total number of plants tested in the experiment noted in parenthesis, if provided; otherwise, a (?) is shown. Genbank accession of infectious clones is provided if available; otherwise, variant information is provided. For methods, see (Fig. 3). Agrobacterium strains are written without abbreviations: OD_600_—optical density at 600 nm; cv.—cultivar.

## Conflict of interest

The authors have no conflict of interest to declare that is relevant to the content of this article.

## Data Availability

Data sharing is not applicable to this article as no new datasets were generated or analysed during the current study.
